# Curcumin improves growth factors expression of bovine cumulus-oocyte complexes cultured in peritoneal fluid of women with endometriosis

**DOI:** 10.18502/ijrm.v16i12.3683

**Published:** 2019-01-28

**Authors:** Hendy Hendarto, Muhammad Yohanes Ardianta Widyanugraha, Widjiati Widjiati

**Affiliations:** ^1^Department of Obstetrics and Gynecology, Faculty of Medicine, Airlangga University/Dr Soetomo Hospital Surabaya, Indonesia.; ^2^Laboratory of Embriology, Faculty of Veterinary Medicine, Airlangga University, Surabaya, Indonesia.

**Keywords:** *Curcumin*, * Bovine*, * GDF9*, * Kit Ligand*, * TNFα*, * Endometriosis.*

## Abstract

**Background:**

Peritoneal fluid (PF) from infertile women with endometriosis contains inflammatory mediators that may interfere with folliculogenesis.

**Objective:**

The aim was to evaluate the effects of curcumin on growth factors expression by evaluating Growth Differentiation Factor-9 (GDF-9), Kit Ligand (KitL), and Tumor Necrosis Factor α (TNFα) expressions in bovine cumulus-oocyte complexes (COC)s cultured with PF from infertile women with endometriosis.

**Materials and Methods:**

In this experimental study, 21 infertile women (aged between 20 and 40 years) who refered to Dr Sutomo Hospital from January to July 2015 were enrolled. COCs were aspirated from antral follicles of bovine ovaries. PF was collected from infertile women with endometriosis undergoing laparoscopy for infertility evaluation. Curcumin, a strong anti-inflammatory turmeric, was added in Tissue Culture Medium 199 (TCM199) and PF for culture medium. Bovine COCs were cultured into three groups of the medium: 1. TCM199, 2. TCM199 + PF, and 3. TCM199 + PF + curcumin. GDF-9, KitL, and TNFα expressions were examined using immunohistochemistry technique.

**Results:**

GDF-9 expression of bovine COCs cultured in PF with curcumin addition (2.67 ± 0.98) was found to increase compared to those cultured without curcumin (0.50 ± 0.67) (*p* ≤ 0.001). It was similar to KitL expression of bovine COCs cultured with curcumin (2.67 ± 1.23), which increased compared to those without curcumin (0.33 ± 0.49) (*p* ≤ 0.001). A significant difference in TNFα expression was noted between groups with or without curcumin (*p* ≤ 0.001).

**Conclusion:**

In the culture of PF from infertile women with endometriosis, curcumin addition improves the growth factors expression of bovine COCs. The increase of GDF-9 and KitL expressions will improve folliculogenesis.

## 1. Introduction

Endometriosis is one of the most common gynaecological diseases characterized by the growth of endometrial-like tissue outside the uterus that subsequently induces a chronic inflammatory reaction (1, 2). Numerous symptoms are associated with endometriosis, including dysmenorrhea, pelvic pain, dyspareunia, and infertility, as well as reduced quality of life (2–4). The relationship between endometriosis and infertility remains unclear. One of the postulations of decreased fertility in women with endometriosis is the reduction of oocyte quality caused by abnormal folliculogenesis related to peritoneal fluid (PF) inflammation and oxidative stress (5–8).

Folliculogenesis is a dynamic process marked by proliferation and differentiation of granulosa cells and maturation of oocyte. The regulation of ovarian folliculogenesis, determined by a number of growth factors and classic endocrine mechanism, provides an optimal environment to produce fertilizable oocyte (9, 10). There are two important growth factors that contribute to the regulation of ovarian folliculogenesis including the interactions between oocyte and granulosa cells. Those growth factors are Growth Differentiation Factor 9 (GDF-9) produced by oocyte, which is useful for granulosa cell proliferation and differentiation, and Kit Ligand (KitL) secreted by granulosa cells, which induces oocyte maturation (11–14).

In women with endometriosis, ovaries are naturally bathed in PF that is rich in inflammatory mediators. This may cause abnormal folliculogenesis and subsequently result in infertility. The PF of women with endometriosis contains a variety of inflammatory mediators, including Tumor Necrosis Factor α (TNFα), Interleukin-6 (IL-6), and Interleukin-8 (IL-8) (15–17).

Curcumin, which is derived from Curcuma longa (turmeric), has a strong potential anti-inflammatory activity. It has been widely used both traditionally and scientifically to treat various conditions including inflammatory diseases such as rheumatoid arthritis, chronic anterior uveitis, and ulcerative colitis through various intracellular and extracellular molecular pathways (18–20). Treatment with curcumin can reduce implant size and cell proliferation in endometriosis model rat (21), but the effects of curcumin on bovine ovarian growth factors in endometriosis and infertility remain controversial.

The objective of our study was to evaluate whether curcumin could improve growth factors expression in bovine cumulus-oocyte complexes (COC)s by analyzing GDF-9, KitL, and TNFα expressions in culture medium with PF from infertile women with endometriosis.

## 2. Materials and Methods

In this laboratory experimental study, two growth factors expression of folliculogenesis regulation on bovine COCs cultured in three different types of medium was evaluated. Group 1 (control group) Tissue Culture Medium 199 (TCM199) only, Group 2 (endometriosis group) TCM199 plus PF from infertile women with endometriosis, and Group 3 (endometriosis+curcumin) TCM199 plus PF from infertile women with endometriosis added with curcumin. A total of 21 bovine COCs were cultured in the medium; each group contained 7 COCs.

### Endometriosis peritoneal fluid (PF)

PF samples were collected from 21 women aged between 20 and 40 years with endometriosis undergoing laparoscopy for infertility evaluation at Dr Sutomo Hospital from January to July 2015. The diagnosis of endometriosis was made by visual inspection and peritoneal biopsy according to the American Society for Reproductive Medicine criteria (22, 23). PF has been collected by aspiration from posterior cul-de-sac during the laparoscopic procedure. The samples were placed in a tube and centrifuged at 600 g for 10 min. The supernatants were stored at –80ºC until analysis.

### Curcumin

Curcumin has been obtained from Merck Schuchardt OHG (85662 Hohenbrunn, Germany). Curcumin 0.2 ml/medium (curcumin 20 μ/ml dissolved into Carboxymethyl Cellulose Sodium/ Na-CMC) was added and homogenized in 10 ml of TCM199 medium and PF from infertile women with endometriosis (30 μl). BSA (3%) was added until the pH reached to 7.4–7.8. The solution was then filtered through a 0.22-μm microfilter, and 100 mL of solution was placed in a Petri dish for culture.

### Bovine cumulus-oocyte complex

COCs aspirated from antral follicles with a diameter of 3–8 mm were obtained from bovine ovaries in a slaughterhouse. The ovaries were washed and stored in 0.89% NaCl with penicillin-G (1000 IU/ml) and streptomycin sulfate (0.2 ug/l) at a temperature of 30–35ºC. Before follicle aspiration, the diameter of the follicles was measured with a caliper. The COCs were aspirated using an 18-G needle connected to a 5-ml syringe containing 1 ml of phosphate buffered saline (PBS) with 3% bovine serum albumin (BSA) and 50 ug/ml gentamycin.

The COCs were washed three times successively in PBS medium and one time in TCM199, placed in TCM199 medium with 50 mIU/ml FSH and 50 mIU/ml LH, divided into three groups by placing them into medium groups 1, 2, and 3, and then incubated at a temperature of 38ºC in 5% CO${}_{2}$ for 24 hr. Subsequently, the COCs of each group were fixated in a glass flask and subjected to immunohistochemical staining for GDF-9 (Bioss Antibodies Inc USA, catalog no. Bs-4720R), KitL (Abcam USA, catalog no. ab52603), and TNFα (Bioss Antibodies Inc USA, catalog no. Bs-2081R) expressions.

The three expressions were semi-quantitatively assessed according to the modified Remmele method which is the result of multiplication between the percentage score of immunoreactive cells (positive cells) with the color intensity score generated on the cell (24).

### Ethical consideration

The study has been approved by the Ethical Committee for Health Research at Dr Sutomo Hospital Surabaya (428/Panke. KKE/X/2014). Written informed consent was obtained from participants before starting the study.

### Statistical analysis

Data analysis was performed using SPSS statistical software (Statistical Package for the Social Sciences, version 17.0, SPSS Inc, Chicago, Illinois, USA). Normality of variable was tested with the Kolmogorov–Smirnov test. One-way analysis of variance (ANOVA) test was used to detect significant differences of all variables. *p* < 0.05 was accepted as statistically significant.

## 3. Results

### GDF-9 expression

**Figure 1:**
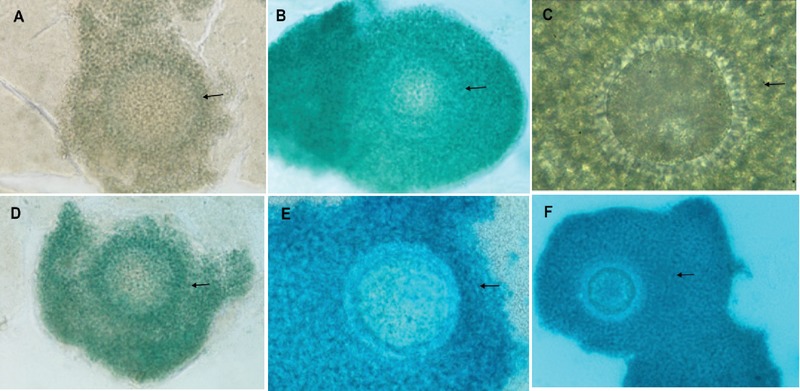
The immunoreactive cells (black arrow) of GDF-9 (A,B,C) and KitL (D,E,F) on immunohistochemical staining of bovine COC in the following medium: 1. control, 2. endometriosis, and 3. endometriosis + curcumin (*p* < 0.05). GDF9 = Growth Differentiation Factor-9; KitL = Kit Ligand; COC = cumulus oocyte complex. Magnification 400x; Olympus BX-50. Pentax optio 230 Digital Camera 2.0 megapixel.

**Figure 2:**
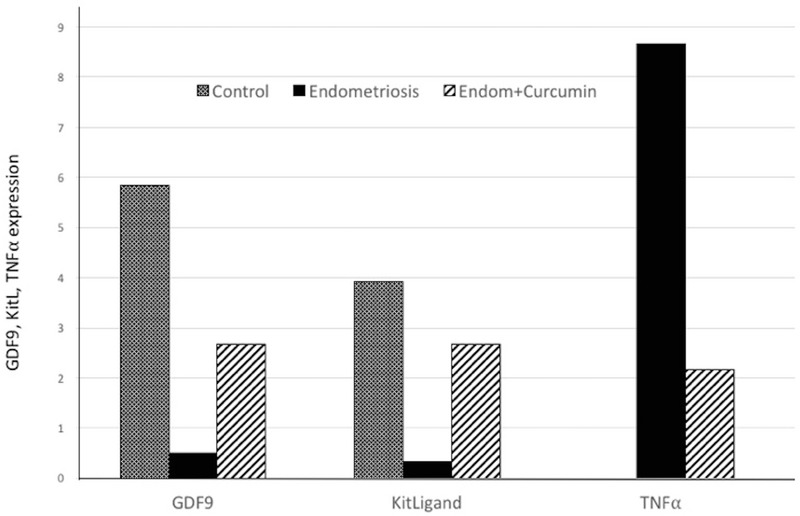
The expression of GDF-9, KitL, and TNF⍺ on bovine COC in the following medium: 1. control, 2. endometriosis, and 3. endometriosis + curcumin (*p* < 0.05).

The GDF-9 expression in bovine COC was determined by the dark color of immunoreactive cells on the immunohistochemical staining result, and it was different among the three groups (Figures 1A, B, C). The mean expression of GDF-9 in bovine COC cultured in PF from infertile women with endometriosis+curcumin (2.67 ± 0.98) increased compared to those cultured without curcumin (0.50 ± 0.67) but reduced compared to the control (5.83 ± 1.58). (*p* ≤ 0.001) (Figure 2).

### Kit ligand expression

The KitL expression in bovine COC was determined by the dark color of immunoreactive cells on the immunohistochemical staining result, and it was different among the three groups (Figures 1D, E, F). The semi-quantitative results of KitL expression in bovine COC were similar to those of GDF-9 expression. The mean expression of KitL in bovine COC cultured in PF from infertile women with endometriosis (0.33 ± 0.49) reduced compared to the control (3.92 ± 2.02) and those in the endometriosis + curcumin group (2.67 ± 1.23) (*p* ≤ 0.001). There was also a significant difference between KitL expression in bovine COC cultured in PF from infertile women with endometriosis + curcumin and control group (*p* = 0.03) (Figure 2).

### TNFα expression

The semi-quantitative results of TNFα expression in bovine COC cultured in control, endometriosis, and endometriosis+curcumin groups were 0.00 ± 0.00; 8.67 ± 3.72; and 2.17 ± 1.69, respectively (25). TNFα expression in bovine COC cultured in PF from infertile women with endometriosis increased compared to those in control group, whereas TNFα expression in bovine COC cultured in PF from infertile women with endometriosis added with curcumin reduced compared to those cultured without curcumin; however, the level increased compared to the control (*p* ≤ 0.001) (Figure 2). In this study, the relationship of three variables was also examined using regression analysis and revealed a significant association among TNFα, GDF-9, and KitL expression cultured in PF from infertile women with endometriosis added with curcumin (*p* < 0.05).

## 4. Discussion

In this study, GDF-9 and KitL expressions in bovine COC cultured with PF from infertile women with endometriosis were significantly reduced compared to control. It suggests that numerous inflammatory mediators in the PF of infertile women with endometriosis enters the bovine COC and further inhibits oocytes and granulosa cells' activity. This results in abnormal folliculogenesis and subsequently infertility.

The PF of infertile women with endometriosis contains a variety of inflammatory mediators (15, 26) and the PF component plays an essential role in the process of folliculogenesis, ovulation, and fertilization. Communication between oocytes and granulosa cells occurs during folliculogenesis to produce mature and fertilizable oocytes. This process is impaired in infertile women with endometriosis. We previously reported abnormal GDF-9 and KitL expression in endometriosis (7, 27).

GDF-9 is part of the TGFβ family member dominantly secreted by oocyte and plays in important role in ovarian folliculogenesis. Study in GDF-9 deficient female mice has resulted in impaired follicular development after primary follicle growth that causes infertility (28). GDF-9 is a determining factor for follicular growth, maturation and survival through inhibition of the apoptosis process in granulosa cell and follicular atresia (29, 30). In this study, the addition of curcumin to the culture medium of PF from infertile women with endometriosis resulted in more improved GDF-9 expression than those without curcumin. It is possible that the anti-inflammatory effects of curcumin release oocyte suppression. By taking into account the fact that the expression of GDF-9 was still lower than the control, it indicates that it is necessary to adjust the dosage of curcumin. These findings indicate that the dose of curcumin plays a role in GDF-9 expression.

In our study, KitL expression in bovine COC cultured in PF from infertile women with endometriosis added with curcumin significantly increased from those cultured without curcumin. It indicates that curcumin has a positive effect on KitL expression. KitL is a growth factor produced by granulosa cells in the ovarian follicle. KitL binds to its receptor in the oocyte, c-Kit, and actively functions for signal pathways during folliculogenesis (31). The initiation of primordial follicle growth, and the development of oocyte and follicle are regulated by the KitL/c-Kit system (13, 31). Study in the buffalo ovaries by Mahajan prove that KitL could be used to predict the oocyte maturation as well as oocyte competence (32).

The changes on two growth factors expression, GDF-9, and KitL suggest that curcumin has a repair effect on oocyte-granulosa cell interactions and the regulation of ovarian folliculogenesis. In order to evaluate that curcumin has a repair effect on oocyte-granulosa cell interactions and the regulation of ovarian folliculogenesis, we assessed the effect of curcumin on bovine COC inflammation via TNFα expression. An increase in TNFα expression was noted in the PF from infertile women with endometriosis compared to that from normal women. In addition, we found a correlation between TNFα concentration and the degree of endometriosis severity. In fact, it has been reported that TNFα plays a role in the inflammatory process and angiogenesis, triggers follicular atresia, and impairs oocyte maturation (33–35). The findings of increased TNFα expressions in bovine COC cultured in PF from infertile women with endometriosis suggest that TNFα from PF enters the COC and is active. The addition of curcumin to the culture media results in an improvement as indicated by the reduced TNFα expression.

Curcumin exhibits anti-endometriosis activities by affecting MMP2 and TIMP2 (36). It works as anti-inflammatory medication by inhibiting cyclooxygenase2 and lipoxygenase and suppressing the activity of NFkB and the level of inflammatory cytokines, including TNFα, IL-6, IL-8, and IFN-γ (18, 19, 20, 36). Based on its mechanism of action, curcumin can suppress an inflammatory process in bovine COC cultured in PF from infertile women with endometriosis. Yet, we found that the decrease in TNFα expression did not reach a normal level. We also found a significant correlation among TNFα, GDF9, and KitL expression, suggesting that the inflammatory factor of TNFα may play a role in GDF-9 and KitL interaction. Studies evaluating the dose-response effects of curcumin on the GDF-9-KitL interaction are still needed.

The limitation of our study included the use of only one concentration of curcumin. However, this was the first attempt to evaluate the effects of curcumin on oocyte-granulosa cell interaction and the regulation of ovarian folliculogenesis in the presence of PF from infertile women with endometriosis.

## 5. Conclusion

Our findings suggest that in the culture of PF from infertile women with endometriosis, curcumin addition improved growth factors expression of bovine COCs through the decrease of inflammation. The increase of GDF-9 and KitL expressions due to the anti-inflammatory effect of curcumin will improve oocyte–granulosa cell interaction and subsequently folliculogenesis. Further studies are warranted.

##  Conflict of Interest

The authors report no conflict of interest.
